# Supplementary material for: Structure Activity Relationship of the Stem Peptide in Sortase A Mediated Ligation from Staphylococcus aureus
Source: Chembiochem. 2022 Sep 20;23(20):e202200412. doi: 10.1002/cbic.202200412 (PMC9632411; doi:10.1002/cbic.202200412)

ChemBioChem

Supporting Information

Structure Activity Relationship of the Stem Peptide in Sortase A Mediated Ligation from *Staphylococcus aureus***

Alexis J. Apostolos, Joey J. Kelly, George M. Ongwae, and Marcos M. Pires*

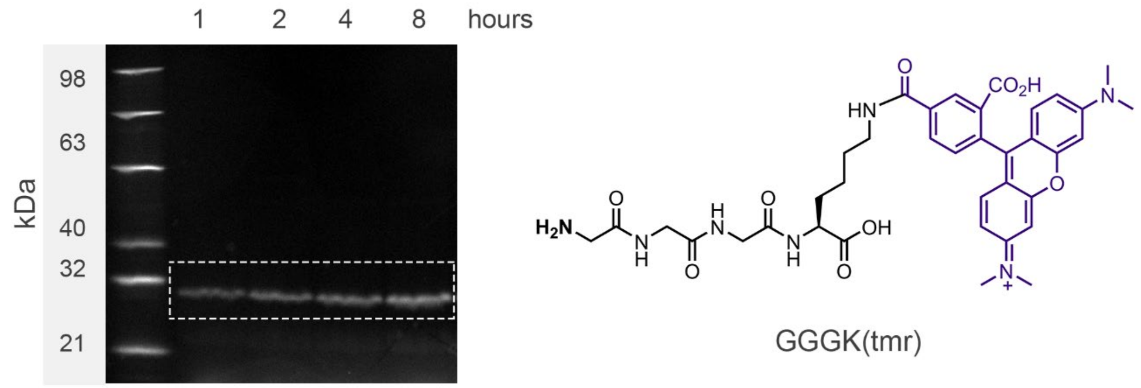

Figure S1. Gel (SDS-PAGE) fluorescence analysis of time points of GGGK(tmr) where the reaction with all elements was quenched at 1, 2, 4, 8 h and displayed an increase in fluorescence as time progressed. An expected product band of ~29 kDa was anticipated for GFP-LPETGGGK(tmr) products.

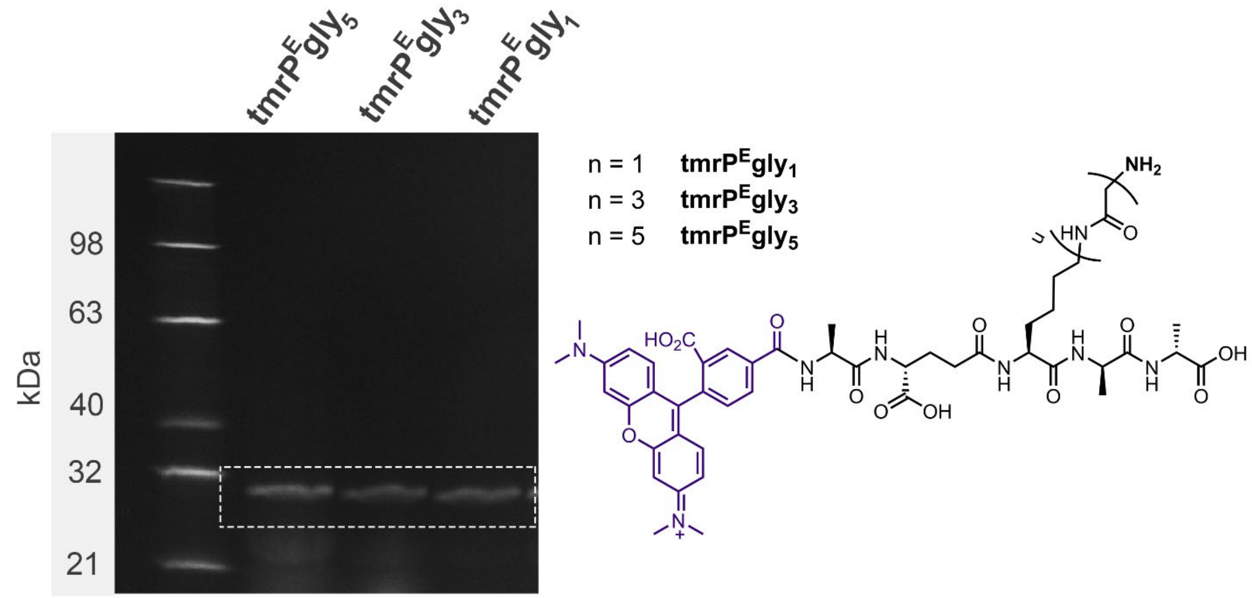

Figure S2. Gel (SDS-PAGE) fluorescence analysis of **tmrP^{E}gly$_5$**, **tmrP^{E}gly$_3$**, and **tmrP^{E}gly$_1$** after incubation of reaction elements for 8 h. Product band is calculated to be ~29 kDa.

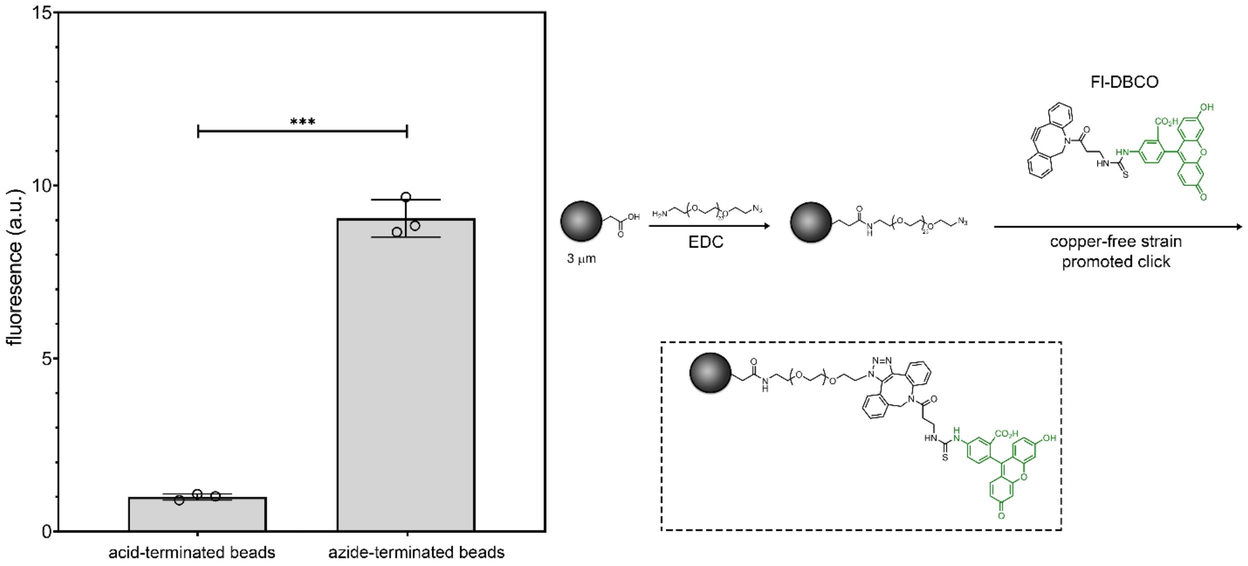

Figure S3. Flow cytometry analysis of H_2N-PEG_{23}-N_3 functionalized beads (or acid-terminated beads as control) treated with DBCO-Fl (25 mm) for 30 min at RT. Beads were washed 3X with 1X PBS before analysis. Data are represented as mean +/- SD (n = 3). *P*-values were determined by a two-tailed *t*-test (* denotes a *p*-value < 0.05, ** < 0.01, ***<0.001, ns = not significant).

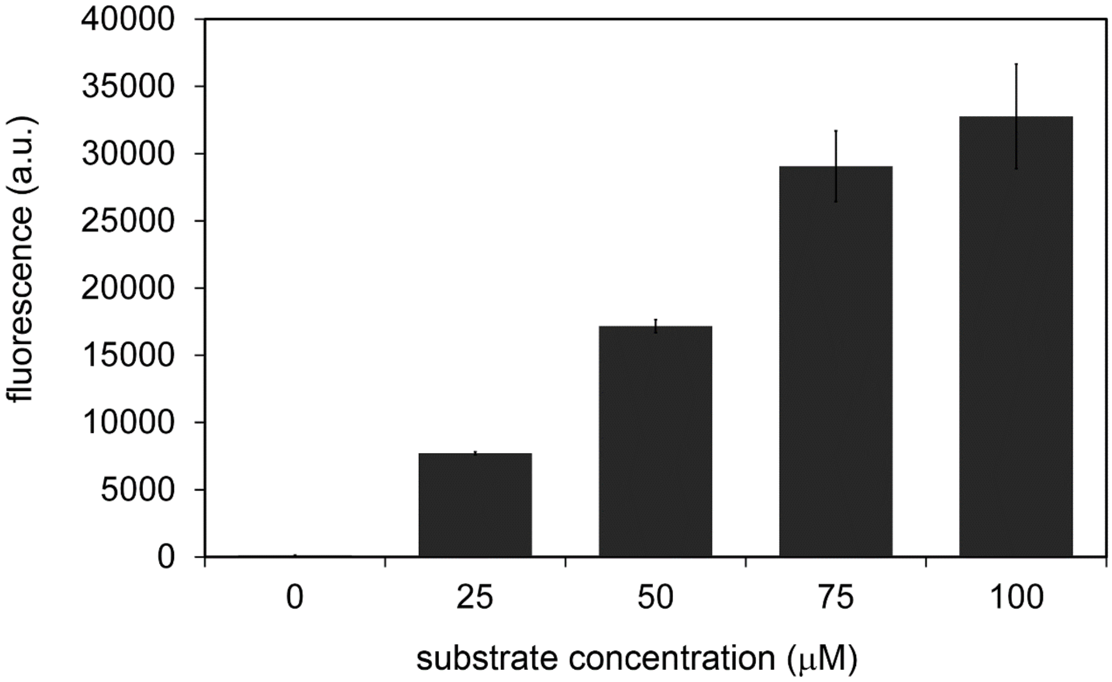

Figure S4. Flow cytometry analysis of sacculi incubated with increasing concentration of Fl-LPMTG in the presence of SrtA. Data are represented as mean +/- SD (n = 3).

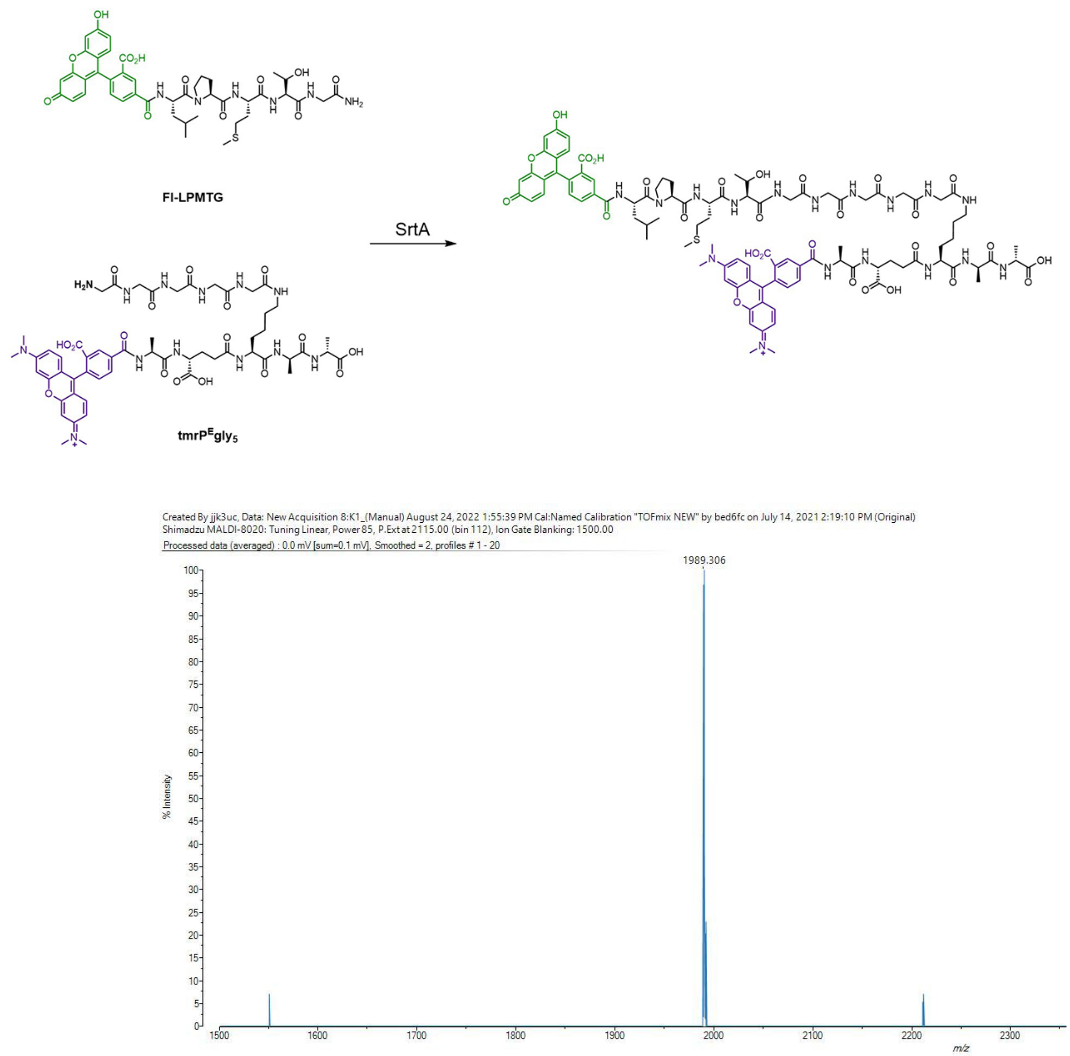

Figure S5. *Top*, reaction scheme for the solution phase SrtA-mediated ligation of the **FI-LPMTG** peptide and **tmrP^{E}gly$_5$**. *Bottom*, MALDI mass spectrum of the reaction showing the expected product (expected mass 1988 Da).

Materials. All peptide related reagents (resin, coupling reagent, deprotection reagent, amino acids, and cleavage reagents) were purchased from ChemImpex. Carboxyl polystyrene beads were purchased from Spherotech. Azido-PEG$_{23}$-Amine was purchased from Broadpharm. Precast gels (10-20% Tricine 1.0 mm) were purchased from Invitrogen.

SrtA in-gel fluorescence Assays. Enzymatic reactions were set up with V_F = 110 μL. In each reaction, 10 μM GFP-LPETG, 10 μM SrtA, 500 μM respective (tmr)peptide, 1X sortase buffer (10X contains 500 mM Tris-HCl, pH 7.5, 1.5 M NaCl, 100 mM CaCl2) were incubated for the time points described. Reactions were quenched with 0.1% TFA. The plasmid for sortase A from *S. aureus* was obtained from Addgene: pET28a-SrtAdelta59 (plasmid no. 51138). The plasmid for GFP-LPETG was also obtained from Addgene (plasmid no. 71754). Each protein was expressed and purified as described by the depositing labs. Invitrogen Novex 10 to 20% Tricine protein precast gels were used to analyze srtA reactions. Each well was loaded with 15 μL of sample and run at 120 V for 75 min. The fluorescent protein standard was purchased from Invitrogen (Cat. No. 10747012) and 5 μL was loaded in each gel. The gel was imaged using a BioRad ChemiDoc XRS+ imager equipped with a XcitaBlue conversion screen with exposure time set to 15 s for all images obtained.

SrtA SPHERO Carboxy Polystyrene Particles Assays. Sphero Carboxy Polystryene beads 3.31 um 5% w/v (Spherotech, 250 μL) were reacted with 0.05 M MES at pH 6 (250 μL), EDC (10 mg), and NH_2-Peg23-Az (BroadPharm, 2 mg) for 24 h. Beads were collected at 3,000g for 15 min. The supernatant was carefully removed, and the beads were washed in PBS. A final working suspension of 5% w/v was made for assays. K(DBCO)LPMTG was reacted with the beads displaying Peg23-Az once the beads were blocked with Tween 80 (0.05%) in PBS, washed, and then resuspended in PBS. The particles were washed as before and resuspended to a 5% w/v suspension. SrtA (10 μM) was incubated with the beads (2.5 μL), 100 μM of each (fl)peptide, and 1X sortase buffer for 4 h at room temperature before the reaction was quenched with 0.1% TFA and washed 3X with 10% SDS. Samples were then diluted 2-fold in PBS and analyzed using an Attune NxT flow cytometer equipped with a 488 nm laser and 525/40 nm bandpass filter. The data were analyzed using the Attune NxT Software, where populations were gated and no less than 10,000 events per sample were recorded.

SrtA Enzymatic Assay with Sacculi. Isolated *S. aureus* sacculi samples were incubated with 20 uM sortase A, 100 uM sorting signal substrate (FL-LPMTG), and 1X sortase buffer (10X contains 500 mM Tris-HCl, pH 7.5, 1.5 M NaCl, 100 mM CaCl2). Samples that contained the covalent inhibitor, MTSET, were run at a concentration of 1 mM. A 10 μL aliquot of the sacculi stock (10 mg/mL) was added to DMF (200 μL) and acetic anhydride (10 μL), DIEA (15 μL) and incubated for 1 h at room temperate. Sacculi was then harvested and resuspended in PBS. All samples were shook at room temperature for 4 h, quenched with 0.1% TFA, and washed 3X with freshly made 8 M urea. Samples were resuspended in a final volume of 200 μL 1X PBS and analyzed by flow cytometry as described above.

Solution phase ligation with SrtA. An enzymatic reaction was set up with VF = 110 mL. In the reaction, 20 μM SrtA, 100 μM **tmrP^{E}gly$_{5}$**, 100 μM FL-LPMTG, and 1X sortase buffer (10X contains 500 mM Tris-HCl, pH 7.5, 1.5 M NaCl, 100 μM $CaCl_2$) were incubated for 2 h. Reaction was quenched with 0.1% TFA. Molecular weight of the conjugated product was confirmed using a Shimadzu MALDI-TOF Mass Spectrometer (MALDI-8020).

Scheme S1. Synthesis of Tripeptides

Fmoc-Lys(Mtt)-Wang resin (250 mg) was added to a 25 mL peptide synthesis vessel with 20% piperidine in DMF for 30 min to remove the base-labile protecting group. The resin was washed with MeOH and DCM (3 x 15 mL each). Fmoc-D-glutamic acid α-amide (3 eq, 193 mg, 2.1 mmol) for "iQ" / "iGln" peptides *or* Fmoc-D-glutamic acid-α-OtBu-OH (3 eq, 223 mg, 2.1 mmol) for "iE" / "iGlu" peptides, HBTU (3 eq, 200 mg, 2.1 mmol), and DIEA (6 eq, 0.183 mL, 4.2 mmol) in DMF (10 mL) were added to the reaction flask and agitated for 2 h at ambient temperature. The resin was washed as before and the Fmoc deprotection and coupling procedure was repeated as before using the same equivalencies with Fmoc-L-Alanine-OH. The Fmoc group of L-alanine was deprotected and coupled with 5(6)-carboxyfluorescein (2 eq, 132 mg, 1.4 mmol) for "FL" peptides or 5(6)-carboxytetramethylrhodamine (2 eq, 151 mg, 1.4 mmol) for "TAMRA" peptides, HBTU (2 eq, 133 mg, 1.4 mmol) and DIEA (4 eq, 0.122 mL, 2.8 mmol) in DMF (10 mL) shaking overnight. The resin was washed as before and the Mtt group of Lys was removed with 15 mL of a 1% TFA solution in DCM with shaking at ambient temperature for 20 min. This step was repeated until a total of 50 mL of 1% TFA was used. Fmoc-Gly-OH (3 eq, 156 mg, 2.1 mmol) was added to the resin with shaking at room temperature for 2 h. The Fmoc group was removed, and this coupling step was repeated 4X to yield a total of 5 Gly on the Lys side chain. The resin was washed as before and added to a solution of TFA/H_2O/TIPS (95%, 2.5%, 2.5%, 20 mL) with agitation for 2 h at ambient temperature. The resin was filtered and resulting solution concentrated *in vacuo*. The residue was trituated with cold diethyl ether. Peptides were purified to homogeneity using RP-HPLC. Purity was assessed using analytical RP-HPLC using a C8 column (detection was performed at 220 nm). Stock solutions were made by dissolving peptides in diH_2O (20 mM final concentration).

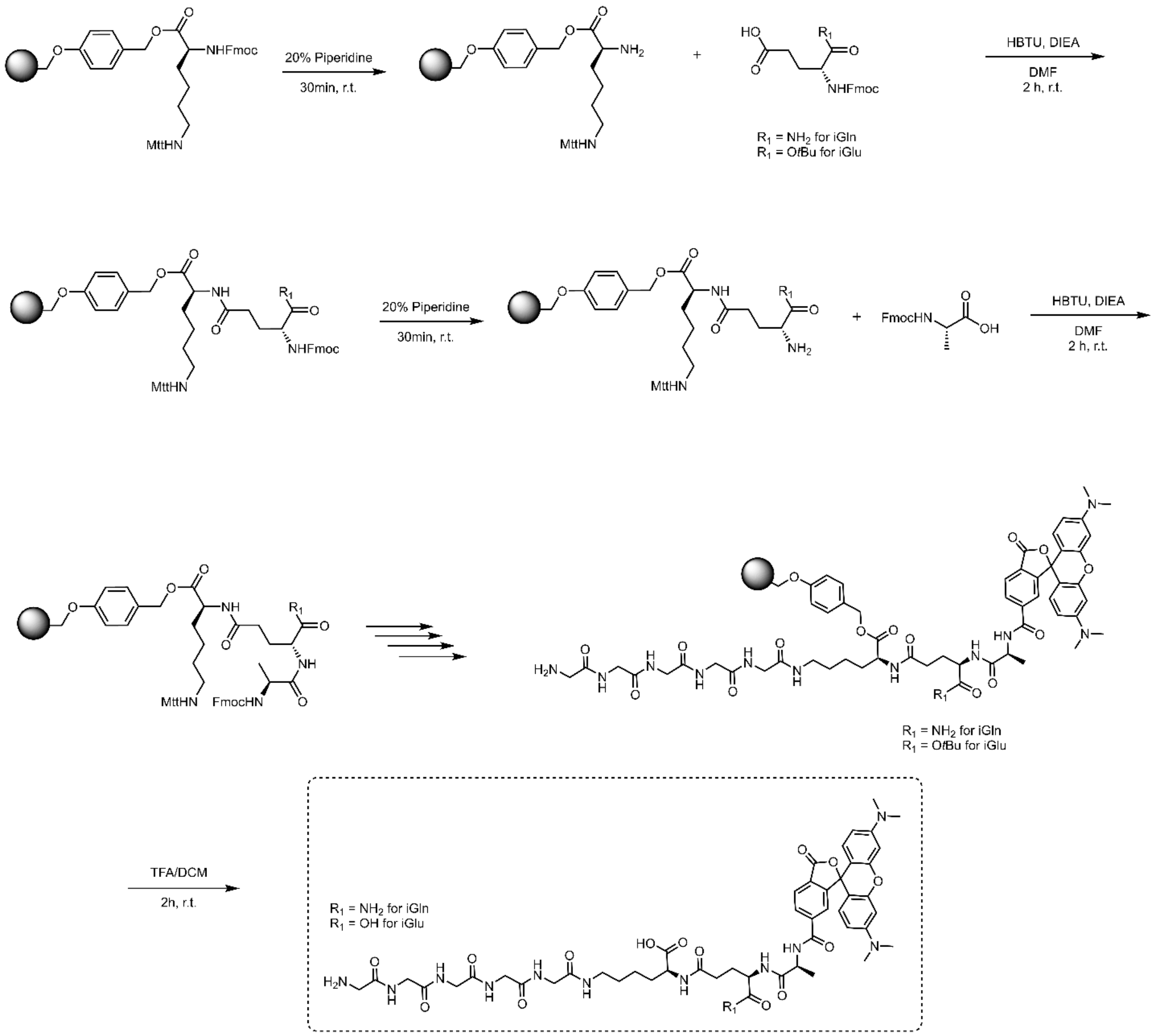
20% Piperidine
30min, r.t.
HBTU, DIEA
DMF
2 h, r.t.
R1 = NH2 for iGln
R1 = OtBu for iGlu
NHFmoc
NH2
MttHN
FmocHN
TFA/DCM
2h, r.t.
R1 = NH2 for iGln
R1 = OH for iGlu

Scheme S2. Synthesis of Tetrapeptides:

Fmoc-D-Ala-Wang resin (250 mg) was added to a 25 mL peptide synthesis vessel with 20% piperidine in DMF for 30 min to remove the base-labile protecting group. The resin was washed with MeOH and DCM (3 x 15 mL each). Fmoc-L-Lys(Mtt)-OH (3 eq, 328 mg, 2.1 mmol), HBTU (3 eq, 200 mg, 2.1 mmol), and DIEA (6 eq, 0.183 mL, 4.2 mmol) in DMF (10 mL) were added to the reaction flask and agitated for 2 h at ambient temperature. The resin was washed as before, Fmoc deprotected, and Fmoc-D-glutamic acid α-amide (3 eq, 223 mg, 2.1 mmol) for "iQ" / "iGln" peptides _or_ Fmoc-D-glutamic acid-α-OtBu-OH (3 eq, 223 mg, 2.1 mmol) for "iE" / "iGlu" peptides was added with the same coupling reagents as previously described. The Fmoc deprotection and coupling procedure was repeated as before using the same equivalencies with Fmoc-L-Alanine-OH. The Fmoc group of L-alanine was deprotected and coupled with 5(6)-carboxyfluorescein (2 eq, 132 mg, 1.4 mmol) for "FL" peptides or 5(6)-carboxytetramethylrhodamine (2 eq, 151 mg, 1.4 mmol) for "TAMRA" peptides, HBTU (2 eq, 133 mg, 1.4 mmol) and DIEA (4 eq, 0.122 mL, 2.8 mmol) in DMF (10 mL) shaking overnight. The resin was washed as before and the Mtt group of Lys was removed with 15 mL of a 1% TFA solution in DCM with shaking at ambient temperature for 20 min. This step was repeated until a total of 50 mL of 1% TFA was used. Fmoc-Gly-OH (3 eq, 156 mg, 2.1 mmol) was added to the resin with shaking at room temperature for 2 h. The Fmoc group was removed, and this coupling step was repeated 4X to yield a total of 5 Gly on the Lys side chain. The resin was washed as before and added to a solution of TFA/H_2O/TIPS (95%, 2.5%, 2.5%, 20 mL) with agitation for 2 h at ambient temperature. The resin was filtered and resulting solution concentrated *in vacuo*. The residue was trituated with cold diethyl ether. Peptides were purified to homogeneity using RP-HPLC. Purity was assessed using analytical RP-HPLC using a C8 column (detection was performed at 220 nm). Stock solutions were made by dissolving peptides in diH_2O (20 mM final concentration).

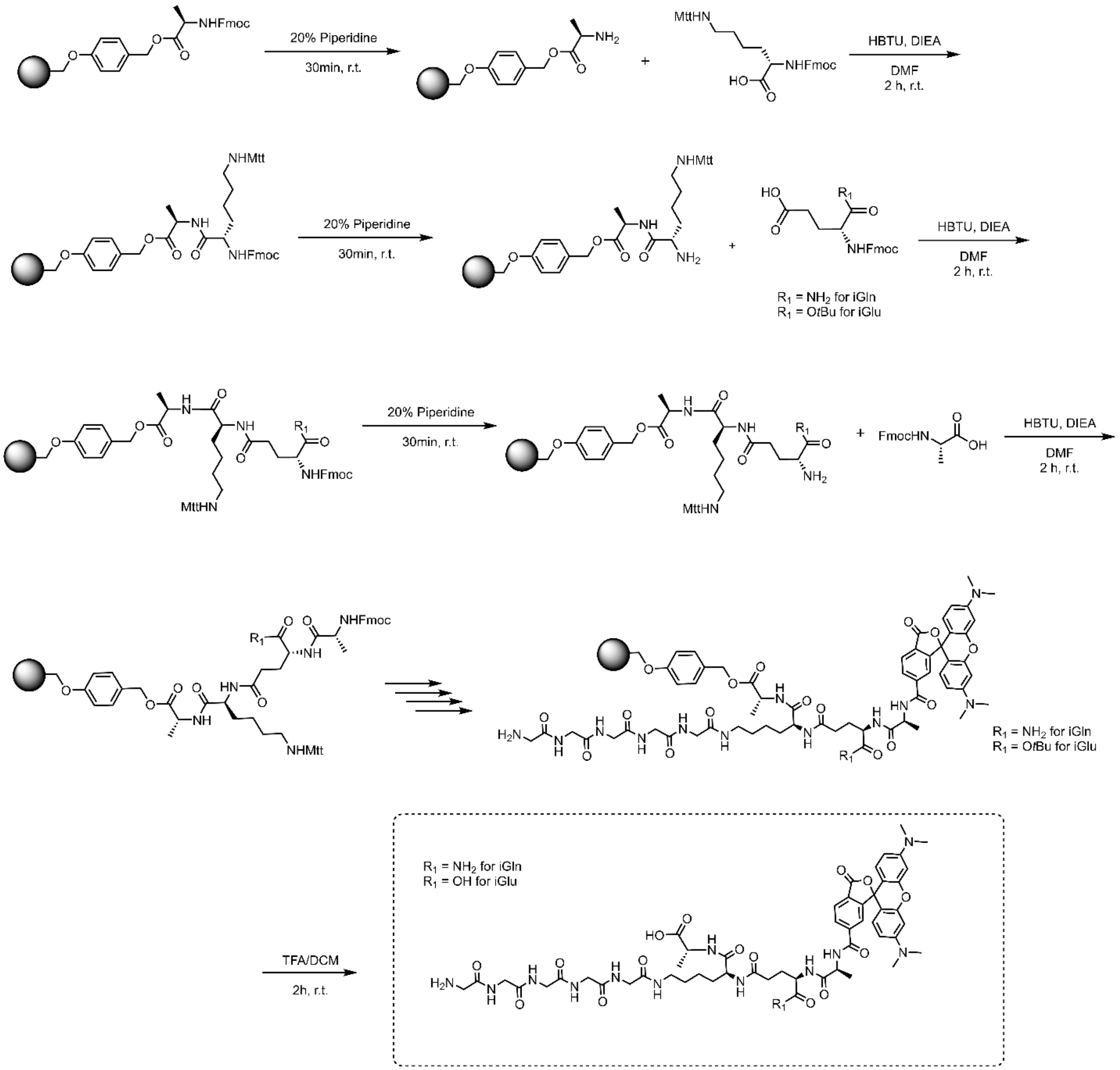
20% Piperidine
30min, r.t.
HBTU, DIEA
DMF
2 h, r.t.
R1 = NH2 for iGln
R1 = OtBu for iGlu
TFA/DCM
2h, r.t.
R1 = OH for iGlu

Scheme S3. Synthesis of Pentapeptides:

Fmoc-D-Ala-Wang resin was added to a 25 mL peptide synthesis vessel with 20% piperidine in DMF for 30 min to remove the base-labile protecting group. The resin was washed with MeOH and DCM (3 x 15 mL each). Fmoc-D-Ala-OH (3 eq, 163 mg, 2.1 mmol), HBTU (3 eq, 200 mg, 2.1 mmol), and DIEA (6 eq, 0.183 mL, 4.2 mmol) in DMF (10 mL) were added to the reaction flask and agitated for 2 h at ambient temperature. The resin was washed, Fmoc-deprotected, and Fmoc-L-Lys(Mtt)-OH (3 eq, 328 mg, 2.1 mmol) with the coupling reagents (as described) were added. The resin was washed as before, Fmoc deprotected, and Fmoc-D-glutamic acid α-amide (3 eq, 223 mg, 2.1 mmol) for "iQ" / "iGln" peptides <u>or</u> Fmoc-D-glutamic acid-α-OtBu-OH (3 eq, 223 mg, 2.1 mmol) for "iE" / "iGlu" peptides was added with the same coupling reagents as previously described. The Fmoc deprotection and coupling procedure was repeated as before using the same equivalencies with Fmoc-L-Alanine-OH. The Fmoc group of L-alanine was deprotected and coupled with 5(6)-carboxyfluorescein (2 eq, 132 mg, 1.4 mmol) for "FL" peptides or 5(6)-carboxytetramethylrhodamine (2 eq, 151 mg, 1.4 mmol) for "TAMRA" peptides, HBTU (2 eq, 133 mg, 1.4 mmol) and DIEA (4 eq, 0.122 mL, 2.8 mmol) in DMF (10 mL) shaking overnight. The resin was washed as before and the Mtt group of Lys was removed with 15 mL of a 1% TFA solution in DCM with shaking at ambient temperature for 20 min. This step was repeated until a total of 50 mL of 1% TFA was used. Fmoc-Gly-OH (3 eq, 156 mg, 2.1 mmol) was added to the resin with shaking at room temperature for 2 h. Some of the resin was split to yield 1 Gly on the Lys side chain. With the remaining resin, the Fmoc group was removed, and this coupling step was repeated 2X to yield a total of 3 Gly on the Lys side chain, or 4X to yield 5 Gly on the Lys side chain. The resin was washed as before and added to a solution of TFA/H_2O/TIPS (95%, 2.5%, 2.5%, 20 mL) with agitation for 2 h at ambient temperature. The resin was filtered and resulting solution concentrated *in vacuo*. The residue was trituated with cold diethyl ether. Peptides were purified to homogeneity using RP-HPLC. Purity was assessed using analytical RP-HPLC using a C8 column (detection was performed at 220 nm). Stock solutions were made by dissolving peptides in diH_2O (20 mM final concentration).

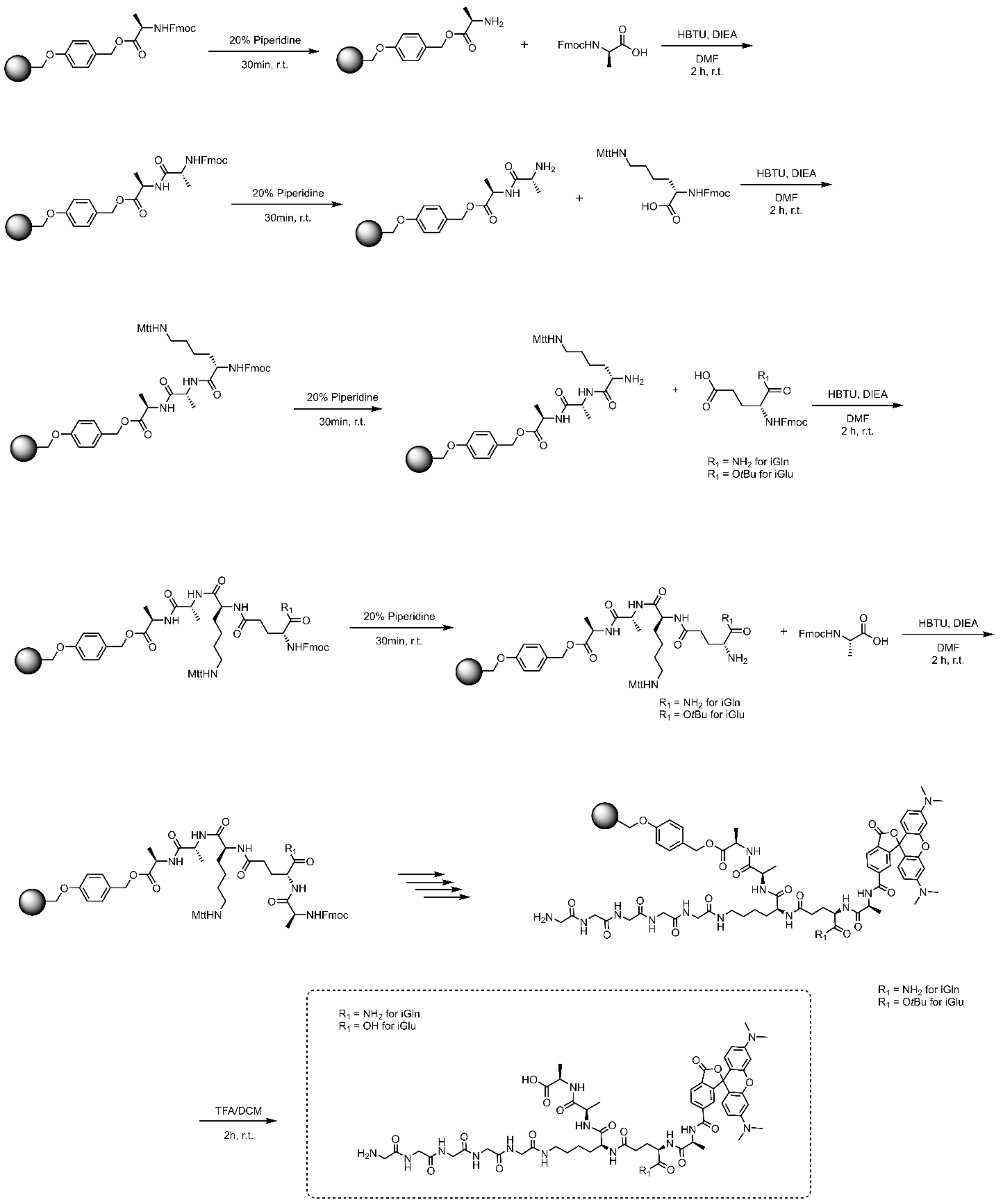
NHFmoc
20% Piperidine
30min, r.t.
NH2
FmocHN
OH
HBTU, DIEA
DMF
2 h, r.t.
MttHN
HO
R1 = NH2 for iGln
R1 = OtBu for iGlu
R1 = OH for iGlu
TFA/DCM
2h, r.t.

Scheme S4. Synthesis of GGGK(TAMRA)

Fmoc-Lys(Mtt)-Wang resin (250 mg) was added to a 25 mL peptide synthesis vessel with 20% piperidine in DMF for 30 min to remove the base-labile protecting group. The resin was washed with MeOH and DCM (3 x 15 mL each). Fmoc-Gly-OH (3 eq, 156 mg, 2.1 mmol), HBTU (3 eq, 200 mg, 2.1 mmol), and DIEA (6 eq, 0.183 mL, 4.2 mmol) in DMF (10 mL) were added to the reaction flask and agitated for 2 h at ambient temperature. The resin was washed as before and the Fmoc deprotection and coupling procedure was repeated as before using the same equivalencies for an additional 2 Gly. After washing, the Mtt group of Lys was removed with 15 mL of a 1% TFA solution in DCM with shaking at ambient temperature for 20 min. This step was repeated until a total of 50 mL of 1% TFA was used. 5(6)-carboxytetramethylrhodamine (2 eq, 151 mg, 1.4 mmol), HBTU (2 eq, 133 mg, 1.4 mmol) and DIEA (4 eq, 0.122 mL, 2.8 mmol) in DMF (10 mL) was added to the vessel with shaking overnight. The resin was washed as before and added to a solution of TFA/H_2O/TIPS (95%, 2.5%, 2.5%, 20 mL) with agitation for 2 h at ambient temperature. The resin was filtered and resulting solution concentrated *in vacuo*. The residue was trituated with cold diethyl ether. Peptide were purified to homogeneity using RP-HPLC. Purity was assessed using analytical RP-HPLC using a C8 column (detection was performed at 220 nm). Stock solutions were made by dissolving peptides in diH_2O (20 mM final concentration).

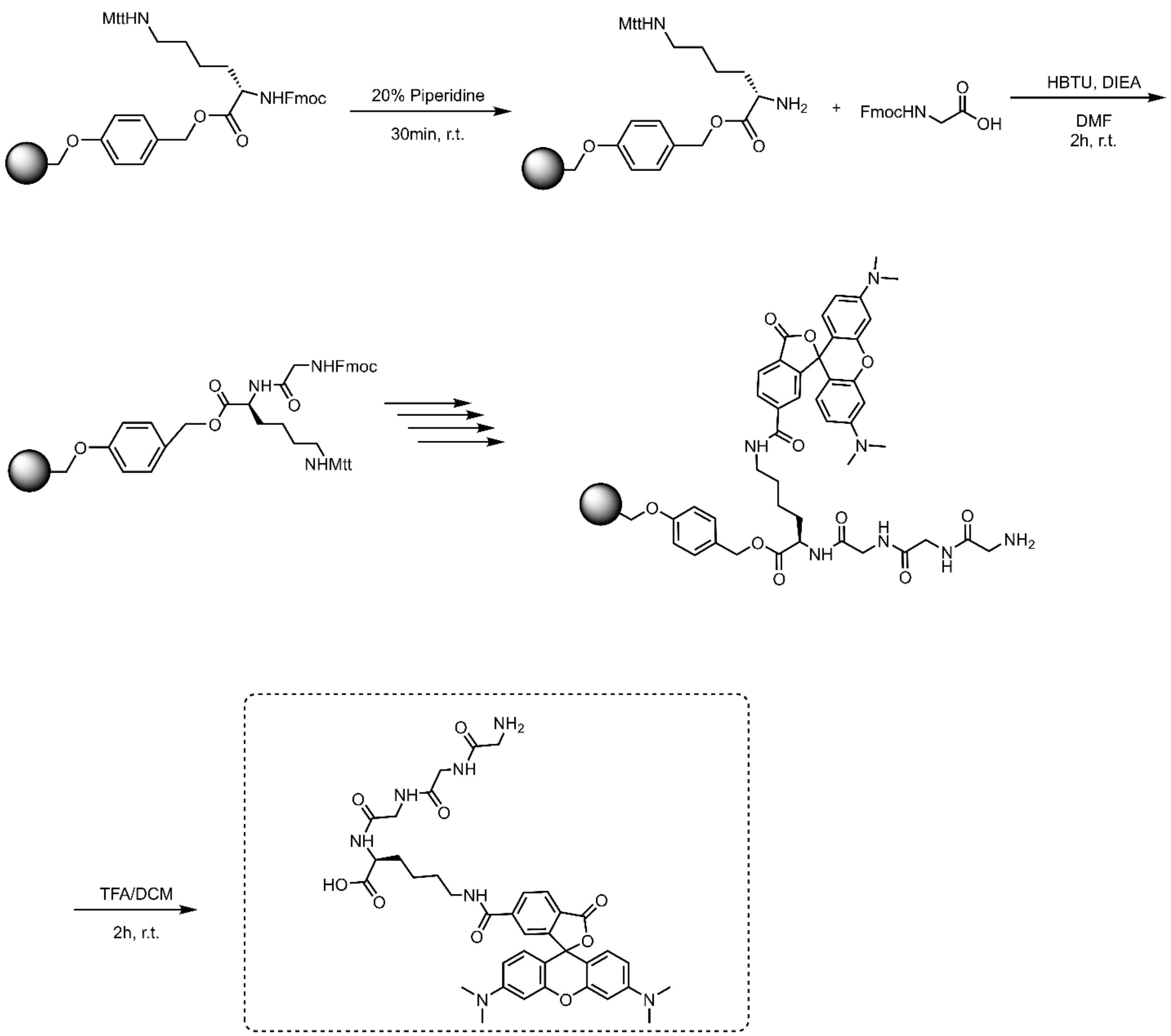

MttHN
NHFmoc
20% Piperidine
30min, r.t.
NH2
+
FmocHN
OH
HBTU, DIEA
DMF
2h, r.t.
NHMtt
TFA/DCM
2h, r.t.
HO

Scheme S4. Synthesis of K(DBCO)LPMTG

A 25 mL synthetic vessel was charged with 500 mg (0.27 mmol) of Fmoc-Rink amide resin. The Fmoc protecting group was removed with a 20% piperidine in DMF solution (15 mL) for 30 minutes at ambient temperature, then washed with MeOH and DCM (3 x 15 mL each). Fmoc-glycine-OH (3 eq, 240 mg, 0.810 mmol), Oxyma (3 eq, 115 mg, 0.810 mmol), and DIC (3 eq, 126 uL, 0.810 mmol) in DMF (15 mL) was added to the reaction vessel and agitated for 2 hours at ambient temperature and washed as previously stated. The Fmoc removal and coupling procedure was repeated as before using the same equivalencies with Fmoc-L-threonine(tBu)-OH, Fmoc-L-methionine-OH, Fmoc-L-proline-OH, Fmoc-L-leucine-OH, and Fmoc-L-lysine(Mtt)-OH. The Fmoc group of L-lysinewas deprotected and resin was capped with acetic anhydride. The Mtt group was removed with 1.5% TFA in DCM for 20 min (5x). Then the lyisine side chain was coupled with DBCO (2 eq, 203 mg, 0.810 mmol), HBTU (2 eq, 201 mg, 0.810 mmol), and DIEA (4 eq, 187 uL, 1.08 mmol) in DMF (15 mL) shaking over-night. The resin was washed as previously described. To remove the peptide from resin, a TFA cocktail solution (95 % TFA, 2.5% TIPS, and 2.5 % DCM) was added to the resin with agitation for 2 hours protected from light. The resin was filtered and resulting solution was concentrated *in vacuo*. The peptide was trituated with cold diethyl ether and purified using reverse phase HPLC using H_2O/MeOH to yield **K(DBCO)-LPMTG**. The sample was analyzed for purity using a Waters 1525 Binary HPLC Pump using a Phenomenex Luna 5u C8(2) 100A (250 x 4.60 mm) column; gradient elution with H_2O/CH_3CN.

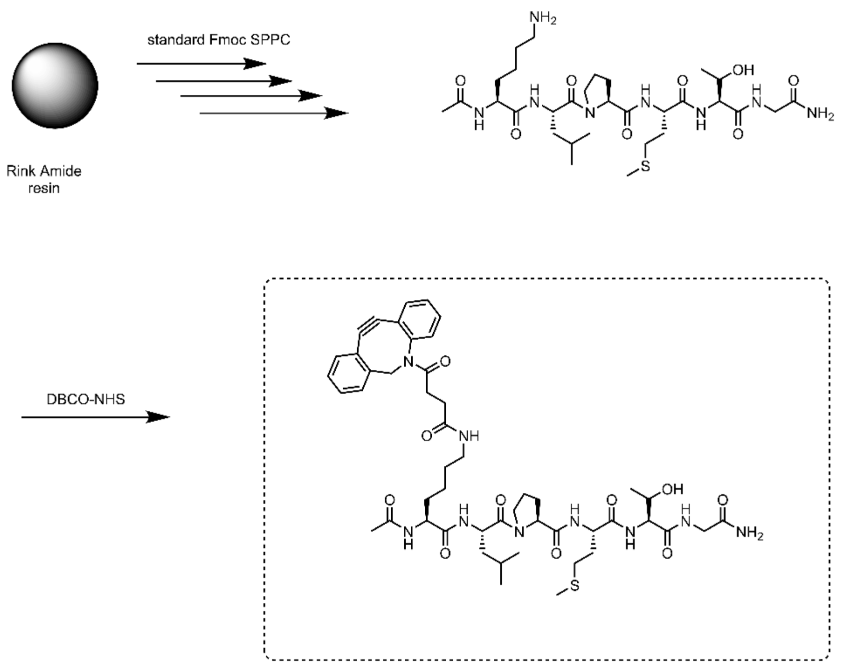

RP-HPLC Analysis:

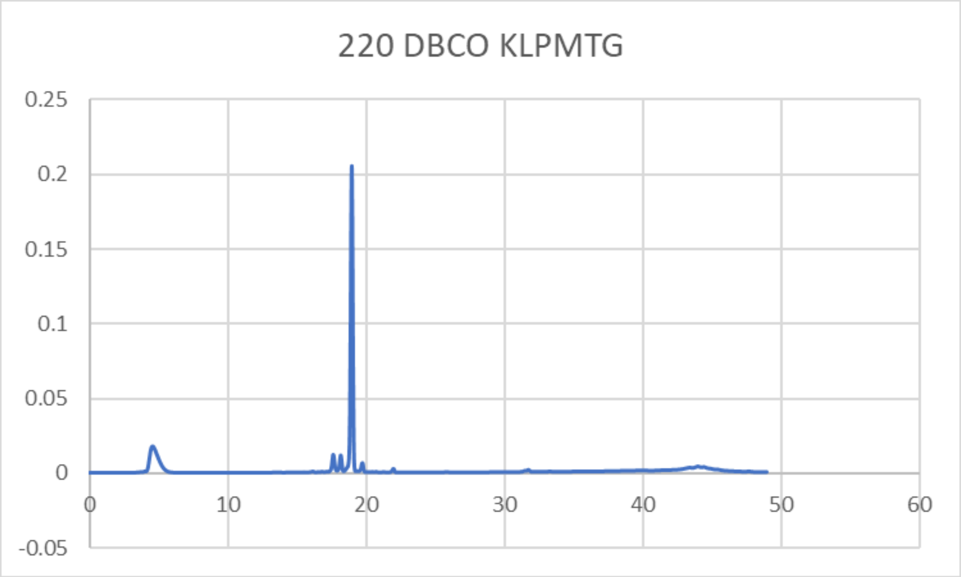

MALDI-TOF:

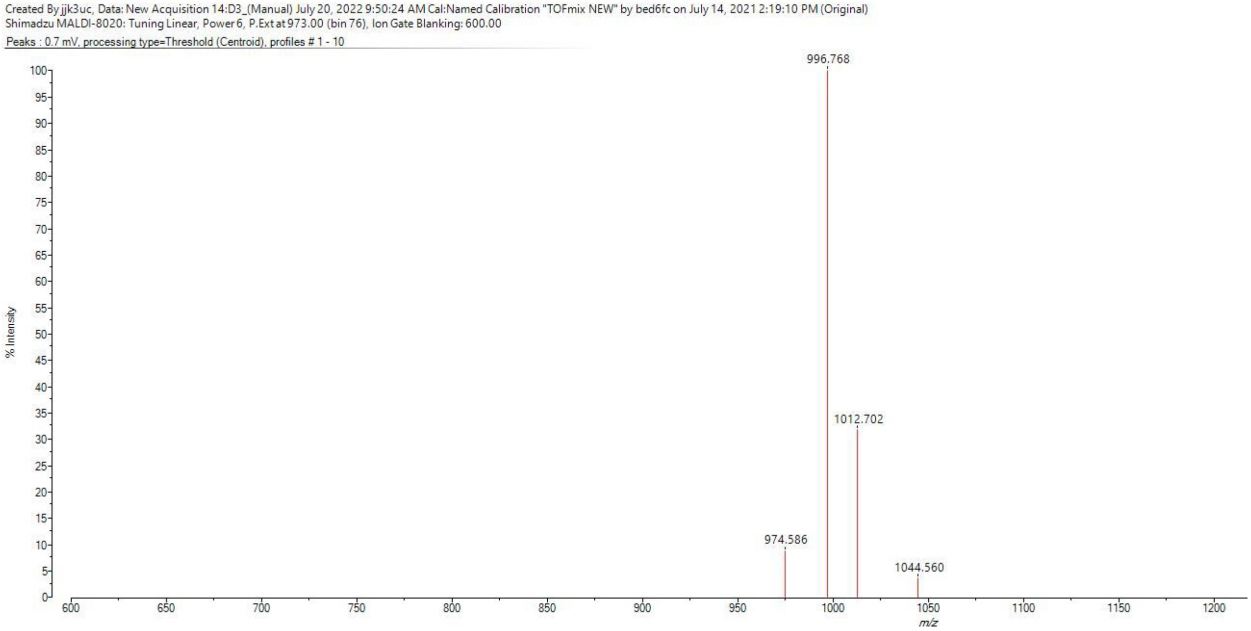

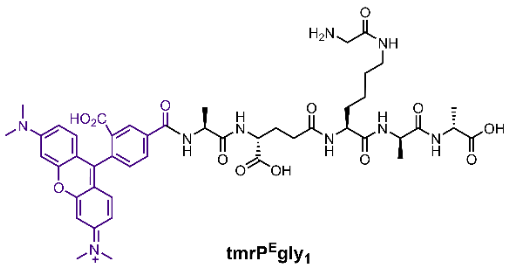

tmrP^{E}gly$_1$

Mass Analysis:

Calculated $[M+H]^{2+}$ = 479.7189
Found = 479.7186

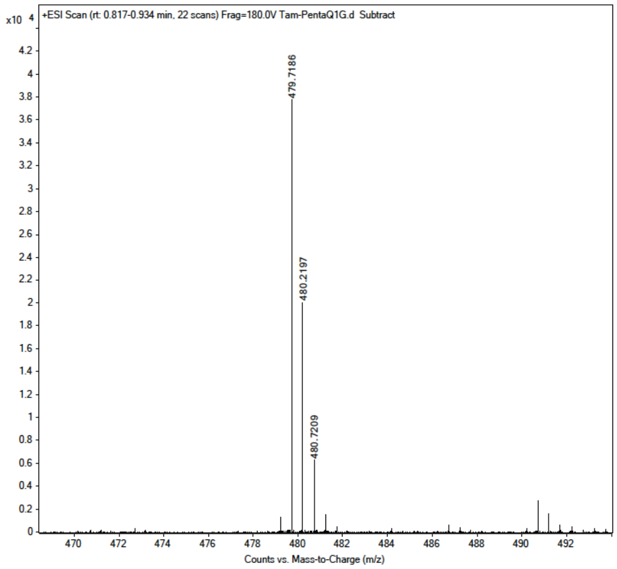

RP-HPLC Analysis:

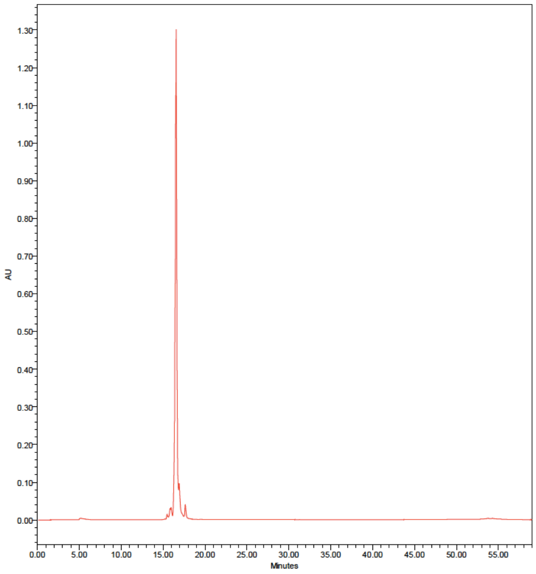

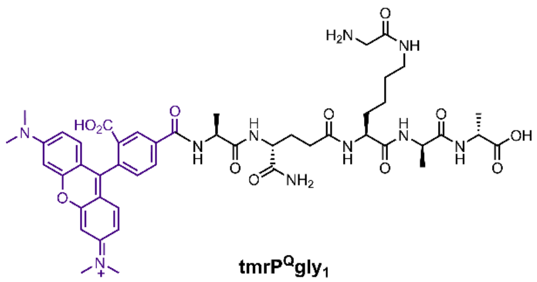

tmrP^{Q}gly$_1$

Mass Analysis:

Calculated $[M+H]^{2+}$ = 479.2269
Found = 479.2266

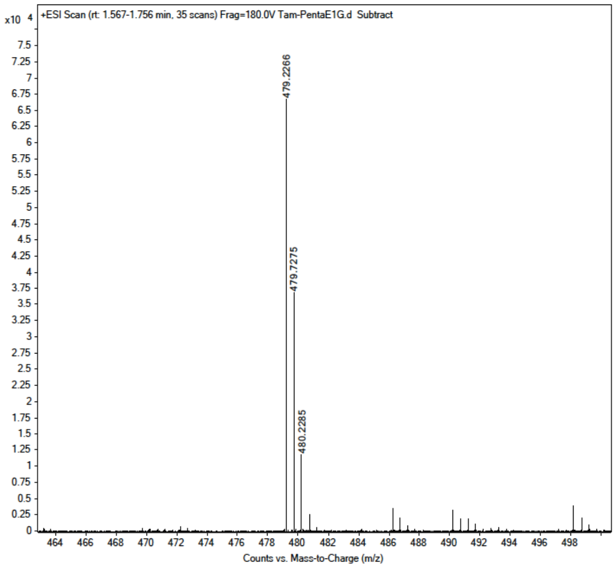

RP-HPLC Analysis:

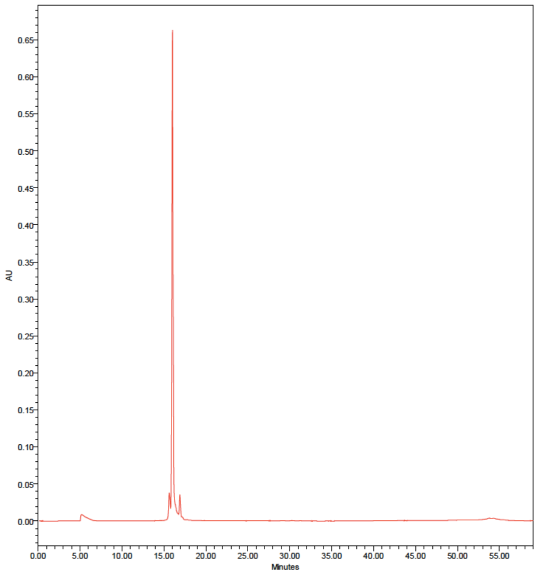

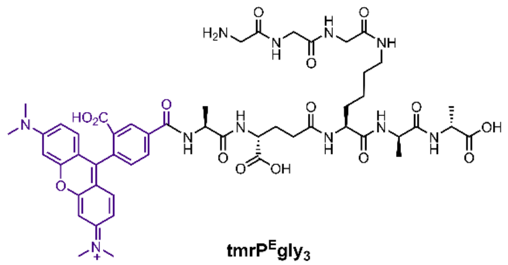

tmrP^{E}gly$_3$

Mass Analysis:

Calculated $[M+H]^{2+}$ = 536.7404
Found = 536.7391

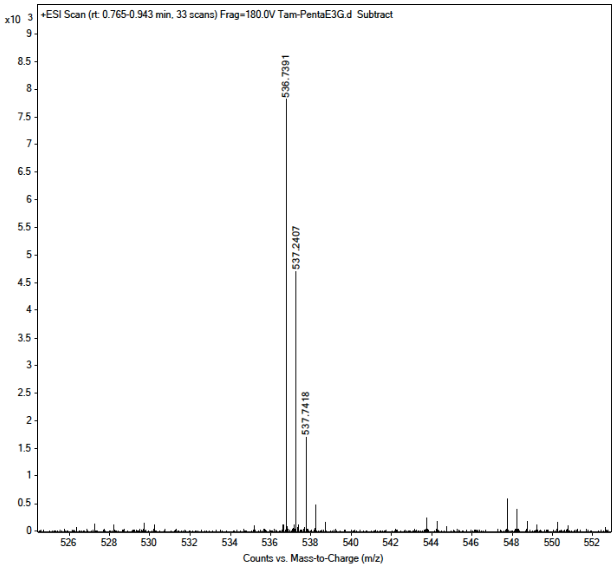

RP-HPLC Analysis:

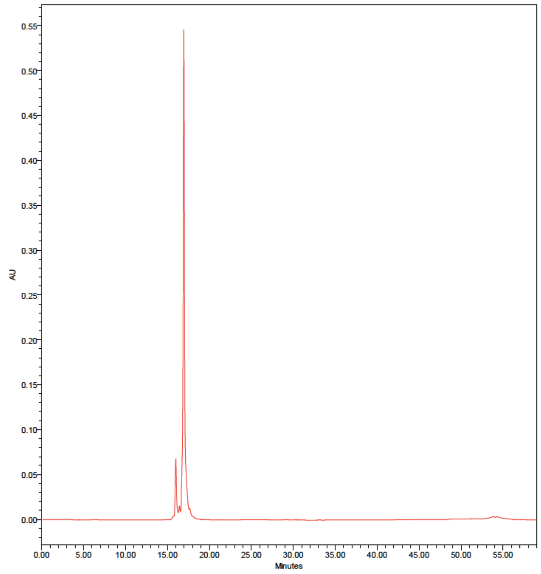

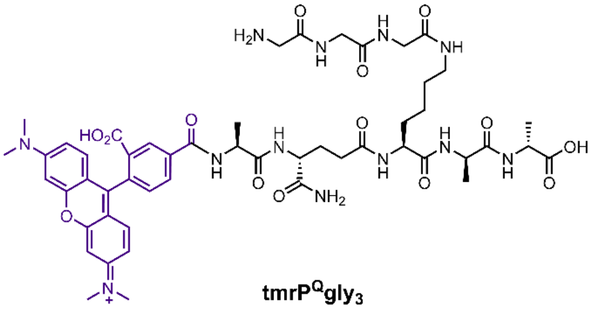

tmrP^{Q}gly$_3$

Mass Analysis:

Calculated $[M+H]^{2+}$= 536.2483
Found = 536.2478

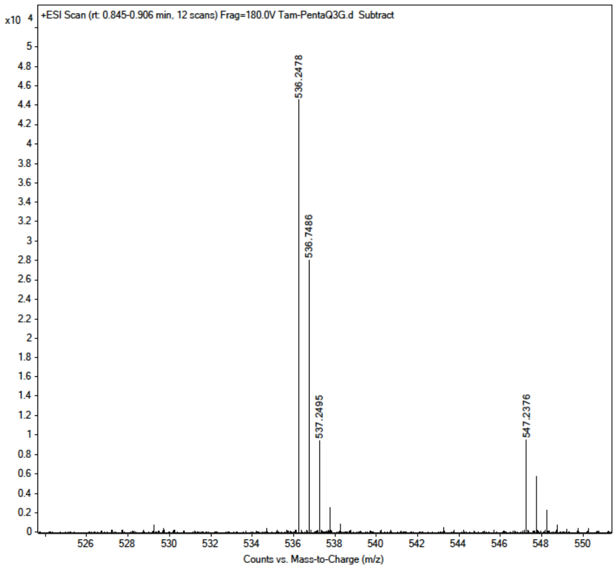

RP-HPLC Analysis:

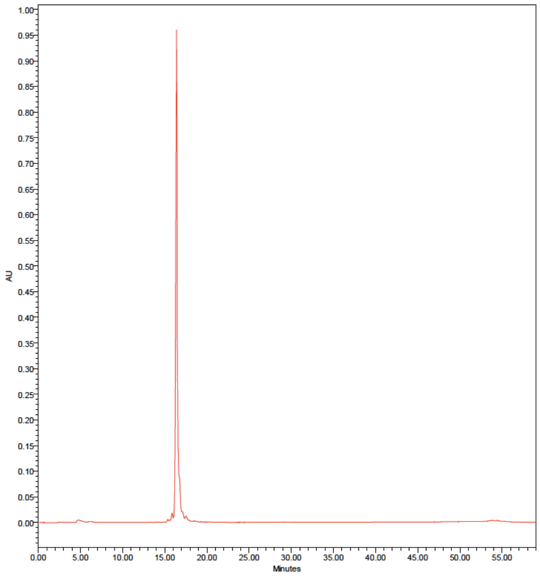

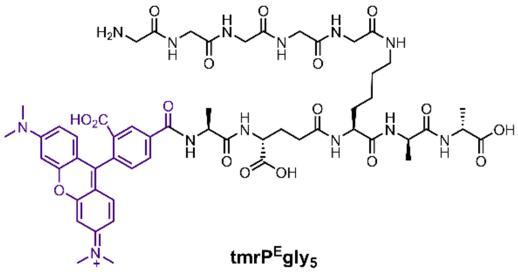

tmrP^{E}gly$_5$

Mass Analysis:

Calculated $[M+H]^{2+}$ = 522.7247
Found = 522.7239

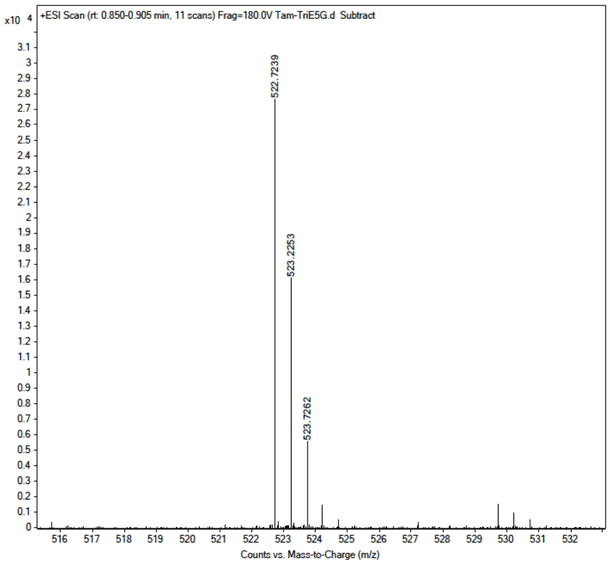

RP-HPLC Analysis:

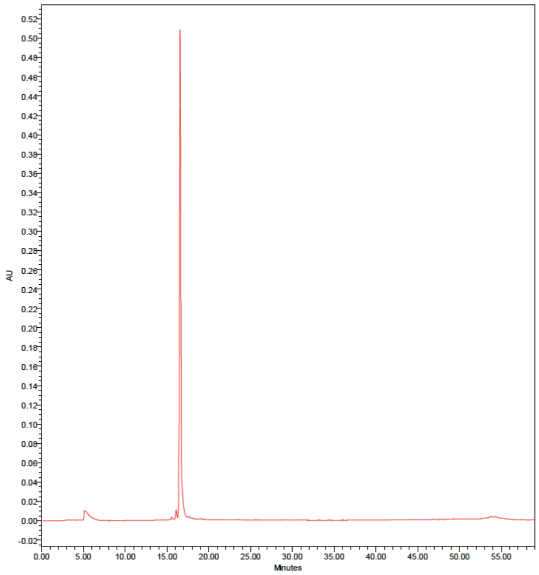

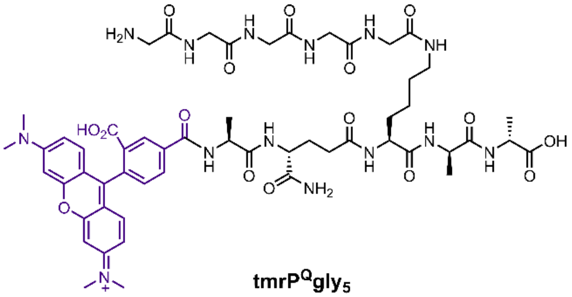

tmrP^{Q}gly$_5$

Mass Analysis:

Calculated $[M+H]^{2+}$ = 522.2327
Found = 522.2314

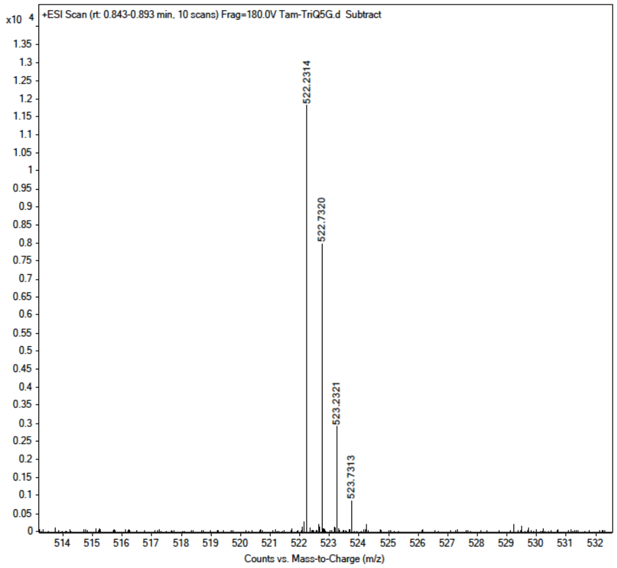

RP-HPLC Analysis:

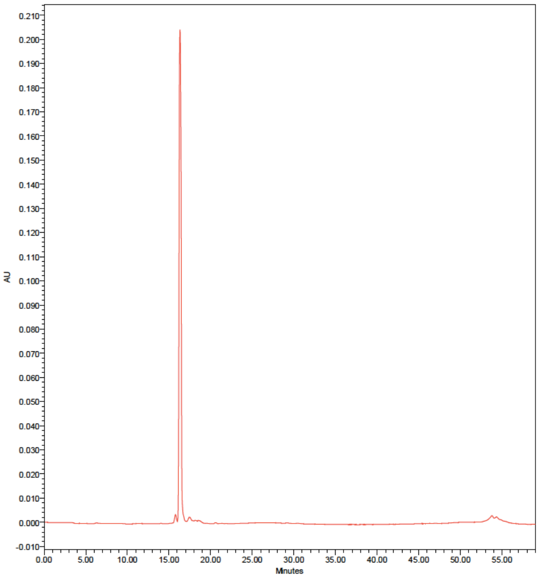

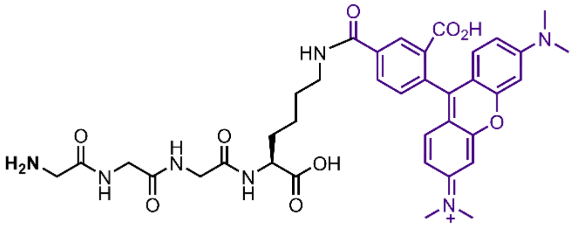

Mass Analysis:

Calculated M+ = 730.3200
Found = 730.3179

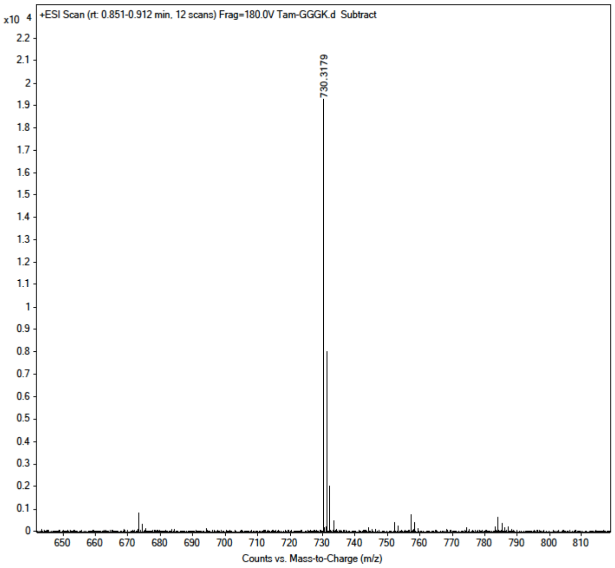

RP-HPLC Analysis:

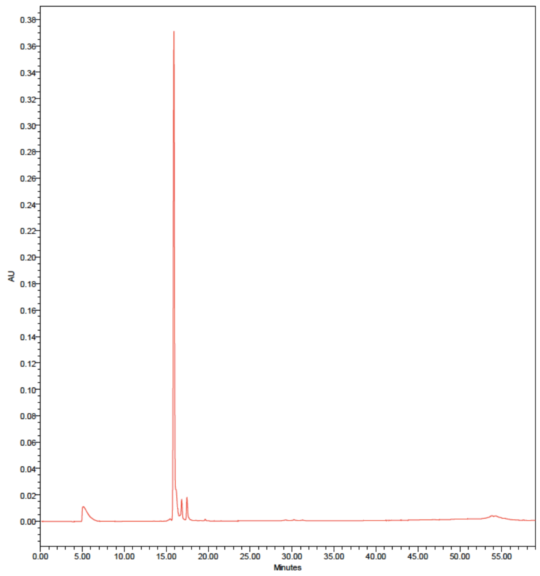

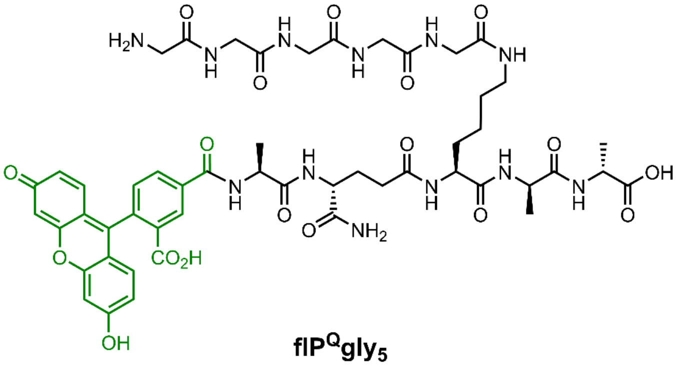

fIP^{Q}gly$_5$

Mass Analysis:

Calculated $[M+H]^+$ = 1132.4223
Found = 1132.4178

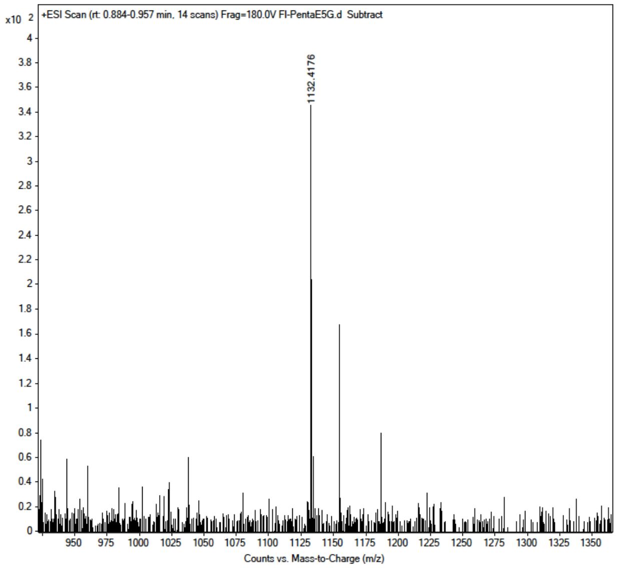

RP-HPLC Analysis:

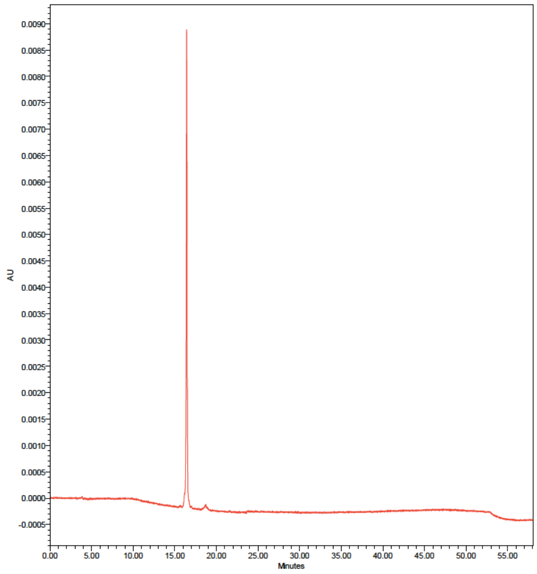

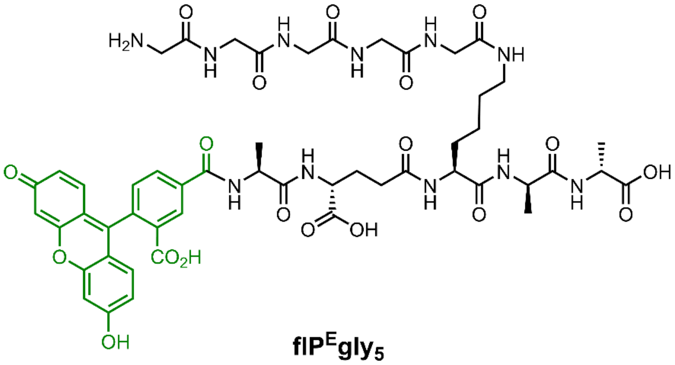

flP^{E}gly$_5$

Mass Analysis:

Calculated $[M+H]^+$ = 1131.4383
Found = 1131.4329

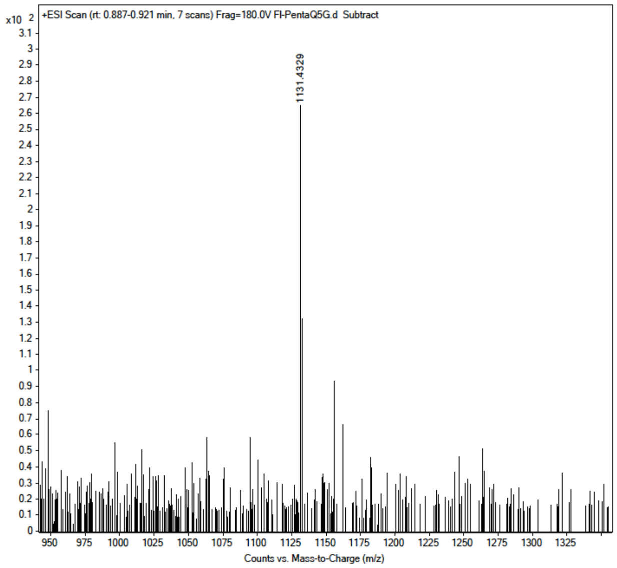

RP-HPLC Analysis:

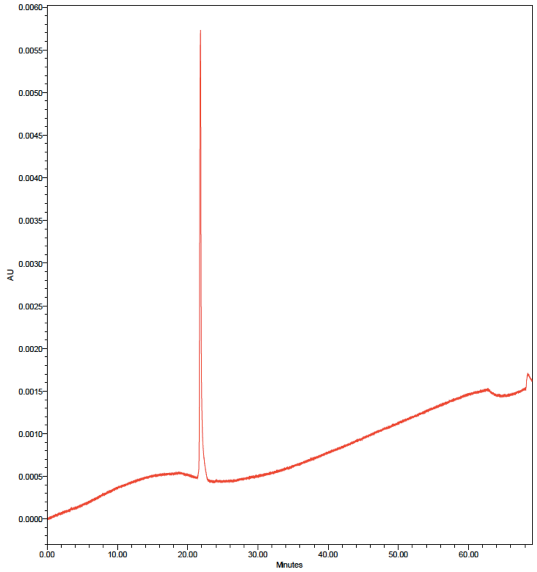

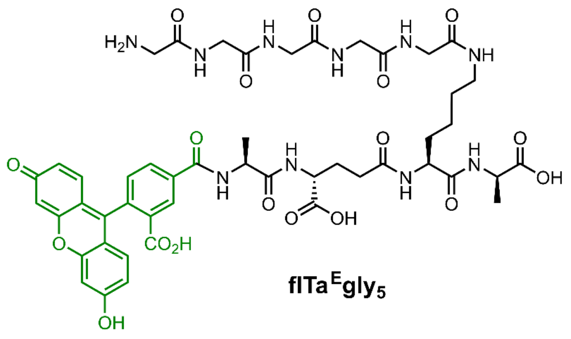

flTa^{E}gly$_5$

Mass Analysis:

Calculated $[M+H]^+$ = 1061.3852
Found = 1061.3718

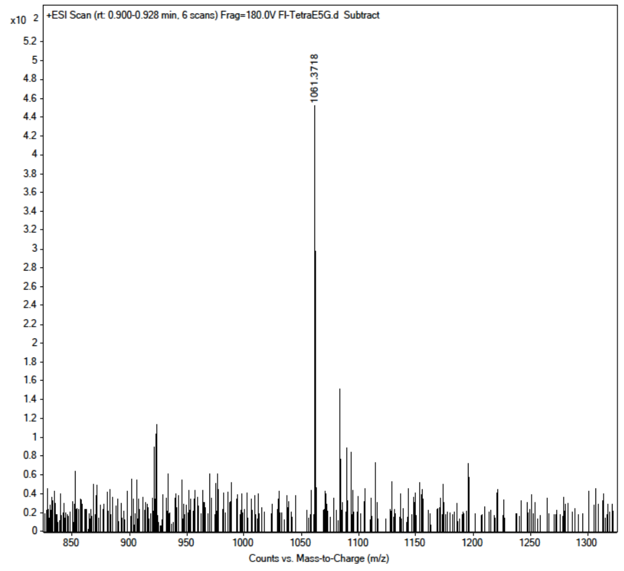

RP-HPLC Analysis:

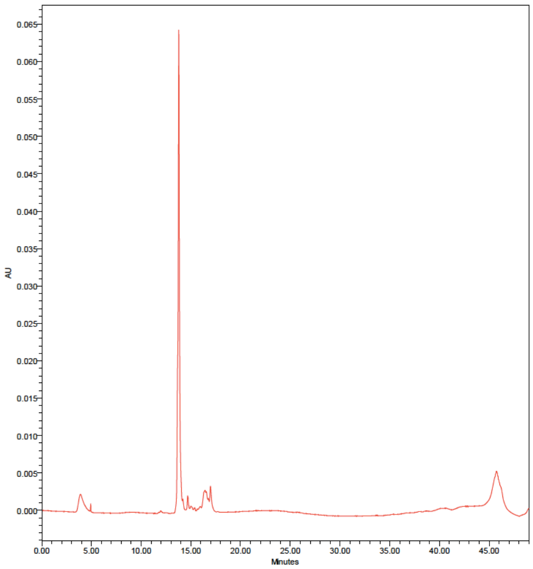

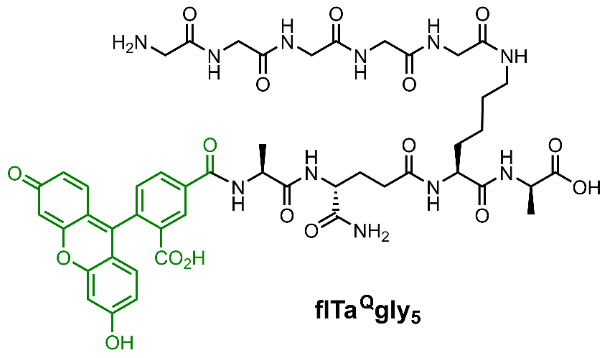

flTa^{Q}gly$_5$

Mass Analysis:

Calculated $[M+H]^+$ = 1060.4007
Found = 1060.3970

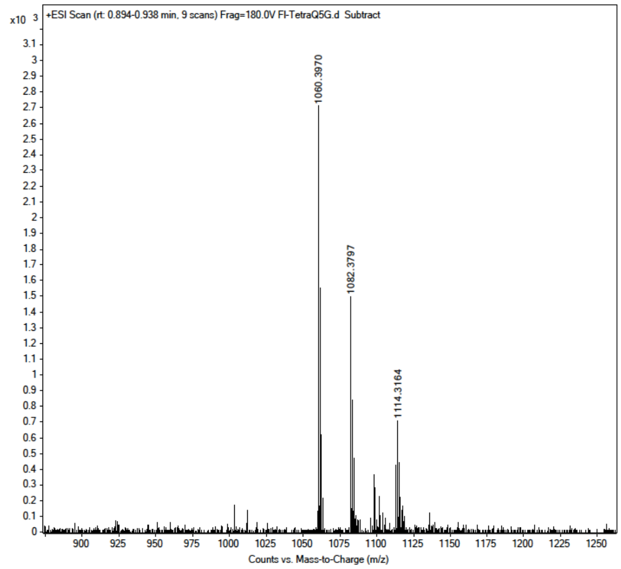

RP-HPLC Analysis:

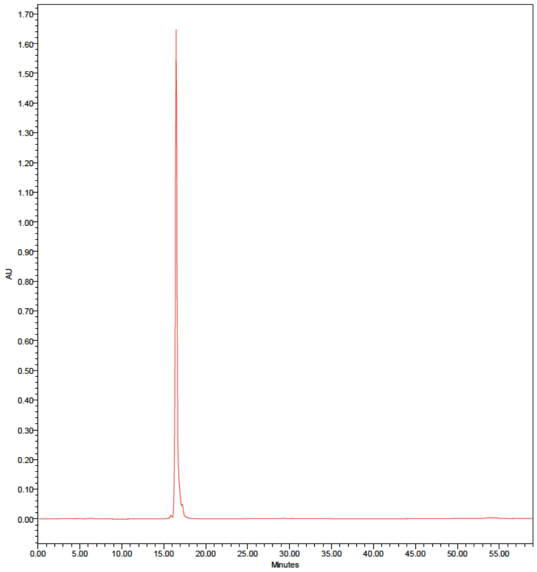

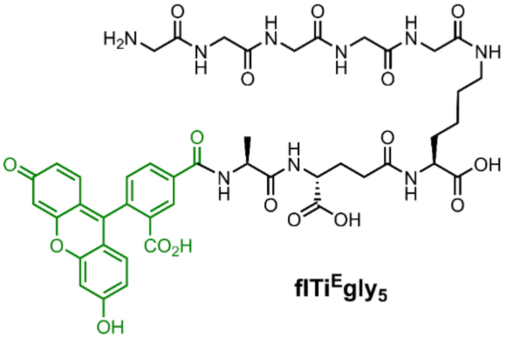

flTi^{E}gly$_5$

Mass Analysis:

Calculated $[M+H]^+$ = 990.3481
Found = 990.3457

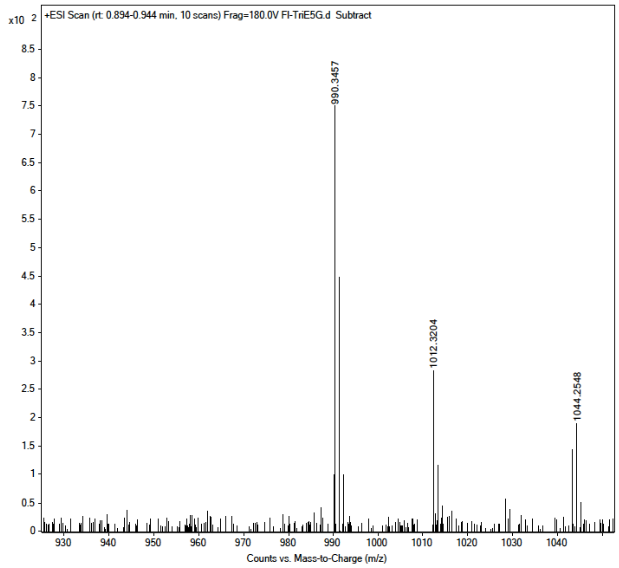

RP-HPLC Analysis:

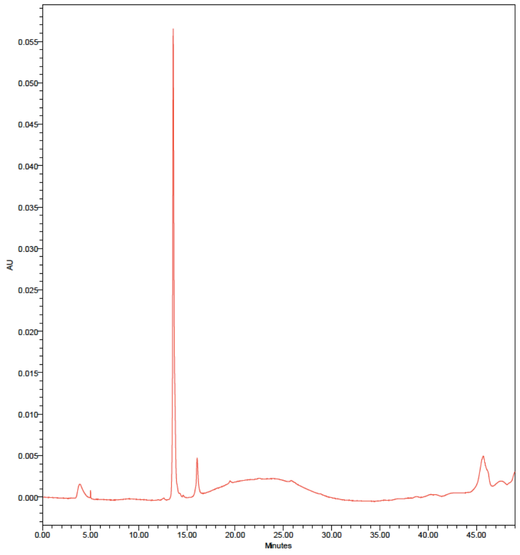

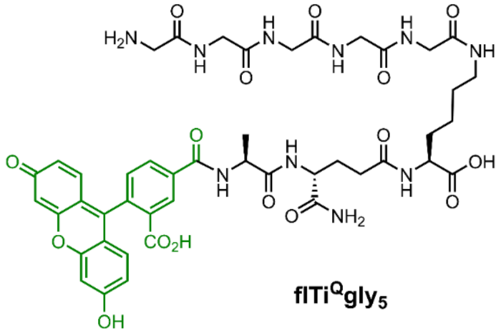

Mass Analysis:

Calculated $[M+H]^+$ = 989.3641
Found = 989.3688

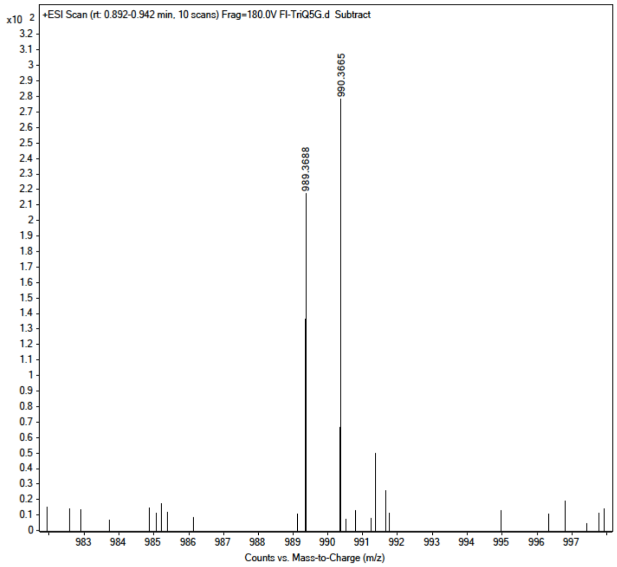

RP-HPLC Analysis:

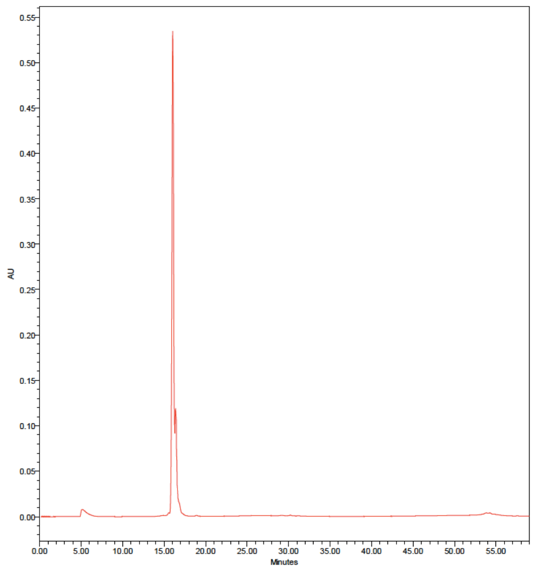

Supplement: Supplementary file 1 — Supporting Information [file CBIC-23-0-s001.pdf]
